# Molecular Imaging of the Dopamine Transporter

**DOI:** 10.3390/cells8080872

**Published:** 2019-08-10

**Authors:** Giovanni Palermo, Roberto Ceravolo

**Affiliations:** Unit of Neurology, Department of Clinical and Experimental Medicine, University of Pisa, 56,126 Pisa, Italy

**Keywords:** [123I]FP-CIT-SPECT, DAT, nigral cells, Parkinson’s disease, parkinsonisms

## Abstract

Dopamine transporter (DAT) single-photon emission tomography (SPECT) with (123)Ioflupane is a widely used diagnostic tool for patients with suspected parkinsonian syndromes, as it assists with differentiating between Parkinson’s disease (PD) or atypical parkinsonisms and conditions without a presynaptic dopaminergic deficit such as essential tremor, vascular and drug-induced parkinsonisms. Recent evidence supports its utility as in vivo proof of degenerative parkinsonisms, and DAT imaging has been proposed as a potential surrogate marker for dopaminergic nigrostriatal neurons. However, the interpretation of DAT-SPECT imaging may be challenged by several factors including the loss of DAT receptor density with age and the effect of certain drugs on dopamine uptake. Furthermore, a clear, direct relationship between nigral loss and DAT decrease has been controversial so far. Striatal DAT uptake could reflect nigral neuronal loss once the loss exceeds 50%. Indeed, reduction of DAT binding seems to be already present in the prodromal stage of PD, suggesting both an early synaptic dysfunction and the activation of compensatory changes to delay the onset of symptoms. Despite a weak correlation with PD severity and progression, quantitative measurements of DAT binding at baseline could be used to predict the emergence of late-disease motor fluctuations and dyskinesias. This review addresses the possibilities and limitations of DAT-SPECT in PD and, focusing specifically on regulatory changes of DAT in surviving DA neurons, we investigate its role in diagnosis and its prognostic value for motor complications as disease progresses.

## 1. Dopamine Transporter Imaging: Role in Diagnosis

**Figure 1:**
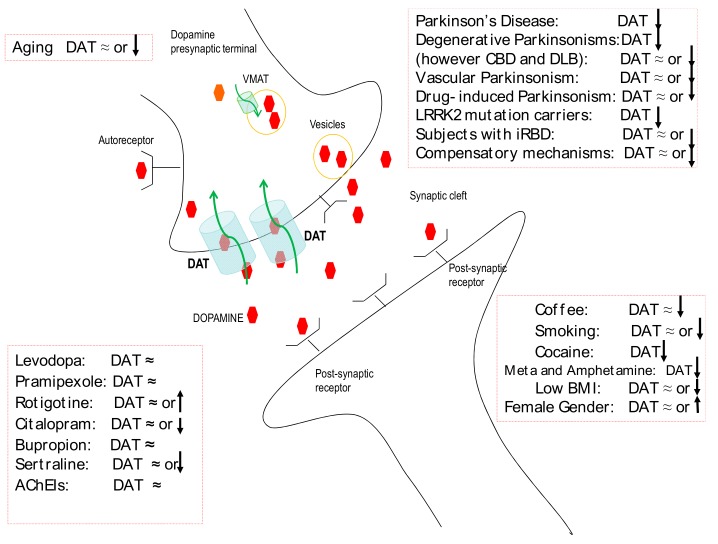
Schematic representation of a dopamine synapse with the dopamine transporter (DAT) and its interference by both external and disease-related factors. (AChEIs: acetylcholinesterase inhibitors; BMI: body mass index; iRBD: idiopathic REM sleep behavior disorder; LRRK2: leucine-rich repeat kinase 2; and VMAT: vesicular monoamine transporter).

Parkinson’s disease (PD) is a progressive neurodegenerative disease characterized primarily by the selective degeneration of dopaminergic neurons in the pars compacta of the substantia nigra (SN). Intraneuronal inclusions composed of aggregates of a-synuclein (α-syn), called Lewy bodies (LBs), are the other neuropathological hallmark [1]. Loss of the nigral neurons, first the lateral tier followed by the medial region, is extensive and characteristic for PD, and it leads to substantial reduction of the presynaptic dopamine transporter (DAT) [2]. DAT is a transmembrane sodium chloride dependent protein expressed only in presynaptic dopaminergic cells. DAT is responsible for reuptake of dopamine (DA) from the synaptic cleft, and it is critical in the spatial and temporal buffering of released DA levels [3]. DAT also has a role in the regulation of quantal DA release at endplates by influencing the DA storage in the synaptic vesicles and the mobility of vesicle pools for release [4,5]. In PD, a lower membrane DAT expression on presynaptic terminals may possibly reflect striatal dopamine terminal loss and is in direct proportion to the magnitude of the depletion of nigral cells. The DAT ligands for single-photon emission tomography (SPECT) have all shown significantly reduced striatal uptake in PD. Abnormal uptake progresses from putamen to caudate and matches, contralaterally, the clinically more affected side, which correlates well with disease severity and duration [6], as well as with both rigidity and bradykinesia, but not with tremor severity [7,8]. [123I]FP-CIT (123I-ioflupane) is one of the most used radiotracers for single-photon emission computed tomography (SPECT) imaging of DAT. Indeed, this [123I]-labelled cocaine analogue shows high specificity for the DAT and has fast kinetics, which allows it to be adequately imaged in clinical practice [9]. Other dopamine transporter ligands that examine dopaminergic systems in vivo include [99mTc]TRODAT [10], [123I] β-CIT [11], and [123I]IPT [12], and the differences among these tracers are mainly in their kinetic properties [13]. Dopamine transporter single-photon emission computed tomography (DAT-SPECT) allows us to evaluate the integrity of the nigrostriatal pathway in vivo, providing the promise of an objective and quantitative biomarker of neuronal degeneration in PD [14]. It is a valuable diagnostic tool to help differentiate essential tremor (ET) from tremor due to parkinsonian syndromes, and it is valuable for a differential diagnosis of degenerative parkinsonisms compared to vascular and drug-induced parkinsonisms [15] (Figure 1). However, the diagnosis of vascular as well as drug-induced parkinsonism might be misleading since evidence of vascular, even strategic, lesions or a positive history of long-term exposure to antidopaminergic drugs does not automatically rule out a concomitant disturbance of the nigrostriatal dopaminergic pathway. Thus, if DAT imaging in pure vascular parkinsonism is typically normal [16], other reports have described an involvement of the nigrostriatal system in patients with parkinsonism and brain vascular lesions [17,18,19]. Similarly, DAT imaging is likely to be normal in pure drug-induced parkinsonism caused by the D2-receptor blockade [20], but involvement of the nigrostriatal system is possible in up to 50% of patients with parkinsonism and with a long-term exposure to antidopaminergic drugs [21,22]. Furthermore, DAT imaging can reveal nigrostriatal impairment, even in isolated/atypical tremors, in which an abnormal SPECT can even predict clinical conversion to a fully blown parkinsonism [23,24,25,26]. All this evidence supports the conclusion that DAT imaging is the most reliable proof of degenerative parkinsonism, and, accordingly, a normal DaTSCAN has been incorporated in the new Movement Disorders Society (MDS) criteria as exclusion criteria for PD [27]. In this respect, cases of parkinsonism without evidence of dopaminergic deficit (so-called scans without evidence for dopaminergic deficit, SWEDD) are not to be considered PD [28], and the term SWEDD should be abandoned, although some data support the notion that an initial, normal DAT-SPECT cannot always exclude early degenerative parkinsonism, suggesting that such patients should be monitored over time [29,30,31]. To complicate matters further, in some degenerative parkinsonisms, such as corticobasal degeneration (CBD) [32] and dementia with Lewy body (DLB) [33,34], in which nigrostriatal dopaminergic dysfunction is usually present, initial DAT-SCANs can be normal, and patients may develop later alterations of DAT imaging in the course of the disease [34,35,36,37,38] (Figure 1).

Some attempts have been performed to use DAT-SCAN for a differential diagnosis between PD and atypical parkinsonisms, such as an evaluation of the asymmetric index and the caudate/putamen ratio, with no significant success in clinical practice [39,40,41]. In visual assessments, a burst striatum pattern was reportedly more common in patients with a clinical diagnosis of atypical parkinsonism, whereas an egg-shaped pattern, which is typical of putaminal degeneration, was reportedly more indicative for PD [42,43]. However, no usefulness on an individual basis has so far been reported in distinguishing PD from atypical parkinsonisms because in pathologically proven atypical parkinsonisms and PD, the DAT-SPECT patterns greatly overlap each other [44].

## 2. Dopamine Transporter Imaging: Drug and Habit Interferences

DAT activity is under the control of different presynaptic proteins, including DA autoreceptors, that can promote both trafficking and the availability of the transporter at the plasma membrane [45] (Figure 1). In animal studies, DAT expression can be interfered by dopaminergic drugs. In rats and monkeys in particular, levodopa can induce an increase of DAT [46,47,48]. However, a decrease of DAT in rats has also been reported with levodopa [49]. Previous studies using DAT radioligands have demonstrated that levodopa appears to have no significant effect on its striatal uptake [50,51]. Similarly, dopamine agonists in standard dosages do not markedly affect the DAT binding capacity independently on the pharmacological profile [52,53]. Current data suggest that D2 autoreceptors induce recruitment of DAT to the membrane, facilitate DA uptake, and DA D2 agonists up-regulate DAT functions in vitro [54]. Recently, the effects of prolonged treatment with DA D3 agonists on DAT have been reported, and they indicated significant underexpression of DAT and the reduction of DA reuptake by dopaminergic neurons [55]. Because of this potential dual effect on D2 or D3 autoreceptors, a trial with pramipexole, a DA agonist, found its use to be ineffective on DAT at the doses usually employed in clinical practice [51]. Instead, the effect of DA D1 agonists on DAT is not entirely known; however, the inhibition of DAT by SKF-83566, a D1 receptor antagonist, has been well documented [56]. After short-term exposure to rotigotine, which has great affinity for D1 and D2 receptors, a mild DAT striatal up-regulation has been recently reported [57]. Based on these findings, PD patients are currently submitted to SPECT studies without the need to withdraw dopaminergic drugs [58].

Selective serotonin reuptake inhibitors (SSRIs) are the mainstay treatment for major depressive disorder, and their effect on DAT is not fully understood yet (Figure 1). Citalopram has been reported to significantly decrease DAT availability, whereas bupropion has no effect [59]. No changes in striatal DAT with different SSRIs have been recently reported [60]. However, some concerns still persist regarding sertraline and citalopram, which in clinical use are preferentially withdrawn before DAT imaging studies. Cholinesterase inhibitors, drugs used for dementia in Lewy body disease and PD with dementia, did not significantly influence DAT expression in a clinical study [61]. Coffee may interfere with dopaminergic transmission, and this action would possibly enhance motor activity and exert an antidyskinetic effect in PD. Nevertheless, in a recent study, chronic coffee consumption was not associated with any significant change in DAT expression [62]. Conversely, cigarette smoking was associated with striatal DAT reduction in PD smokers, as already reported in non-PD smokers [63,64]. However, results of a recent meta-analysis on nicotine did not show any significant effects between smokers and nonsmokers on DAT availability, whereas significant downregulation was reported for most studies investigating the effect of stimulant drugs on DAT such as cocaine, methamphetamine, and amphetamine [65] (Figure 1).

## 3. Dopamine Transporter Imaging: Relationship with Nigral Cell Counts

The density of DAT on presynaptic terminals is considered a surrogate marker for dopaminergic nigral cell counts and vitality, but a clear, direct relationship between nigral loss and DAT decrease has, so far, been controversial. Reduced DAT striatal availability parallels loss of striatal DA and loss of nigrostriatal fibers in monkeys chronically treated with 1-methyl-4-phenyl-1,2,3,6-tetrahydropyridine (MPTP) [66]. Recently, in a cohort of autopsy-confirmed neurodegenerative disease cases, researchers investigated whether changes in DAT images reflected loss of DAT, because of cell death or neurodegenerative pathology, by examining the influence of nigral neuronal loss as well as nigral (α-syn, tau) and striatal (α-syn, tau, amyloid-β) pathology on striatal uptake in 4 cases of Alzheimer’s disease (AD), 7 cases of dementia with Lewy bodies (DLB), and 12 Parkinson’s disease dementia (PDD) cases. Subjects underwent antemortem dopaminergic scanning and postmortem assessments (mean interval 3.7 years). In all striatal regions, tracer uptake was associated with nigral dopaminergic neuronal density but not α-syn, tau, or amyloid-β burden [67]. In another paper, postmortem SN cell counts in patients (PD, DLB, AD, multiple system atrophy, and CBD) who had previously undergone DAT-SPECT were correlated with striatal DAT values. They found a high correlation between striatal uptake binding and postmortem SN cell counts, confirming the validity of DAT imaging as an excellent in vivo marker of nigrostriatal dopaminergic degeneration [68]. In contrast, in nonhuman primates with experimental parkinsonism, striatal uptake of the DAT ligand did not faithfully reflect nigral cell counts throughout the full range of neuronal loss, with a flooring effect once nigral loss exceeded 50% [69]. To support this finding, in a recent study in which nigral neuron numbers were calculated for 18 patients (11 patients had neuropathologically confirmed PD) who had been examined with DAT-SPECT before death, postmortem SN–pars compacta (SNc) neuron counts were not associated with striatal DAT binding in PD. These results fit with the theory that there is no correlation between the number of SN neurons and striatal DA after a certain level of damage has occurred. Striatal DAT binding in PD may reflect axonal dysfunction or DAT expression rather than the number of viable neurons [70]. It should be considered that in these studies there is a possible bias represented by the time interval between the in vivo scan and the postmortem examination (3.5 years in the group with the shorter interval). A possible approach to overcome this limitation might be to correlate in vivo dopaminergic, neuromelanin-rich neurons in SN measured by magnetic resonance imaging (MRI) with DAT ligand striatal uptake. MRI measures of neuromelanin (both area and signal intensity) show decreased levels in the region corresponding to SNc in PD patients, with respect to healthy controls [71,72,73,74,75,76,77,78], and patients with ET [79] that paralleled disease severity and duration [80,81]. Moreover, a good correlation exists between signal intensity in neuromelanin-sensitive MRIs and the density of neuromelanin-containing neurons in the SNc [82,83]. Interestingly, a good correlation between neuromelanin-sensitive MRI SN measures and striatal DAT-SPECT values has been recently demonstrated [84,85].

Direct visualization of the SN is one of the unmet needs in neuroimaging of parkinsonisms, with controversial results obtained from the various attempted approaches [86]. By using susceptibility-weighted imaging (SWI) at 7T and even at 3T MRI, the loss of the hyperintense laminar or ovoid-shaped areas present in SN of healthy controls [87,88], identified as nigrosome-1, has been proposed as a morphological marker of nigral depletion in both PD and atypical parkinsonisms, with no significant difference on an individual basis [89].

These observations could also have implications in the potential detection of the premotor phase of PD in order to evaluate at-risk subjects. In this respect, carriers of dominant PD genes, such as leucine-rich repeat kinase 2 (LRRK2) mutations, or subjects with REM sleep behavior disorder (RBD) are ideal candidates to evaluate the detection of preclinical/premotor PD by means of nuclear medicine or MRI. The potential role of DAT imaging has been demonstrated [90,91,92,93], whereas data on SWI–MRI are only sparse. In a study of a family with three LRRK2 mutation carriers, the DAT-SPECT was abnormal for all family members, whereas the MRI signal in the SN was abnormal only in the unaffected mother and in the affected daughter, and it was normal in the unaffected son [94]. Moreover, in a series of 15 RBD subjects studied by 3T MRI, an alteration of nigrosome-1 was reported in two-thirds of them [95].

Emerging interest has pointed towards the mutual relationship between SN MRI anatomical changes and the assessment of dopaminergic nigrostriatal functions, as assessed by DAT imaging. A good agreement between SN-MRI abnormalities and [123I]FP-CIT-SPECT has been reported [96]. However, preservation of nigrosome-1 was reported in few patients with PD and degeneration of the nigrostriatal pathway. The disagreement between MRI and SPECT was also observed in patients with CBD [97], in a carrier of the LRRK2 mutation [94], and in one patient with RBD [98]. By assuming DAT-SPECT is a surrogate marker of degenerative parkinsonisms, such data could be interpreted as the effect of some inaccuracies of SWI–MRI, but some considerations could also be raised. If iron is assumed to be responsible for SN abnormality in SWI–MRI, it should be noted that iron is not invariably associated with degeneration. The two methods explore nigral neurons at different levels, and DAT-SPECT could overestimate degeneration because of early DAT down-regulation [99], or, alternatively, it might reflect synaptic degeneration prior to neuronal death [100].

At the molecular level, loss of dopaminergic axonal terminals seems to precede SN cell body loss [101]. Specific deregulated pathways linked to axonal degeneration have been found to occur prior to the onset of α-syn pathology in the SN of early PD subjects [102]. Neurodegeneration in PD might begin at the nerve terminal, and neuronal death may result from a “dying back” process [103]. Indeed, α-syn is a natively unfolded presynaptic protein involved in synaptic transmission and synaptic vesicle retrieval, and accumulation of misfolded fibrillar α-syn has been coupled with the severity of neurodegeneration [104]. Thus, the role of axonal destabilization as the putative mechanism that primarily causes neurotoxicity in PD has advanced. Notably, a decrease of DAT not only occurs early in the disease process, but it is greatest in the early stages of PD than in subsequent years, disappearing by year four of diagnosis [105]. In consonance with this, nigral cell loss appears to be profound and substantial soon after diagnosis, and it becomes modest to negligible for decades thereafter, suggesting a nonlinear reduction in tracer binding as well as in the rate of dopaminergic cell loss over the course of the illness [106]. Neuropathological studies in human brain samples, and both in vivo and in vitro models, support the hypothesis that nigrostriatal synapses may be affected at the earliest stages of the neurodegenerative process. Mechanisms leading to either structural or functional synaptic dysfunction are starting to be elucidated, and they include dysregulation of axonal transport, impairment of the exocytosis and endocytosis machinery, altered intracellular trafficking, and loss of corticostriatal synaptic plasticity. Recent data support the view that, in PD, early synaptic dysfunction is directly caused by α-syn oligomers through different and multiple mechanisms [107]. Although α-syn is expressed throughout the brain, DA neurons are the most vulnerable in PD, likely because α-syn directly regulates DA levels as a negative modulator by inhibiting enzymes responsible for its synthesis and by interacting with and reducing the activity of vesicular monoamine transporter 2 (VMAT2) and DAT [108].

## 4. Dopamine Transporter Imaging: A Window of Compensatory Mechanisms

Neuronal loss in the SN, with or without LBs, occurs with normal aging in a spectrum that spans from age-associated brain changes to structural alterations of neurodegenerative diseases like PD [109]. In general, there is abundant evidence in support of the association between aging and PD: the incidence and prevalence of PD strongly increase with age, and aging is the predominant risk factor [110]. PD affects 3–4% of individuals over the age of 65 years and reaches a prevalence of 4% in the highest age groups [111]. Furthermore, age at onset of PD significantly affects the phenotype and accelerated progression of disease, especially in the early–middle phase [112]. However, although there is a marked age-related decline in DA levels (about 7% per decade), aging alone cannot lead to the critical level of cell loss necessary for parkinsonian signs to emerge (estimated as approximately 80% striatal dopamine depletion). Moreover, the patterns of striatal dopamine loss in PD and normal aging are different, affecting mostly the ventrolateral segment of the SN in patients with PD [113]. Similarly, age-related striatal DAT decline does not seem to be exaggerated or accelerated in PD [114], even though activation of regulatory and compensatory mechanisms directed at maintaining DA uptake, superimposed upon disease-related DA terminal loss, may have masked significant age correlations [115,116] (Figure 1). As a consequence of this, currently PD is not merely considered the result of an accelerated aging process, even though the effects of aging on dopaminergic neurotransmission in the striatum might be consistent with a pre-PD state [117].

Similarly, Lewy pathology has been found at autopsy in the brains of elderly individuals without clinical PD in association with intermediate SN neuron loss between PD cases and controls, supporting the concept that subjects with incidental LB pathology represent preclinical PD [118]. Indeed, LB pathology and a nigrostriatal dopaminergic deficit are thought to antedate the clinical diagnosis by several years [119]. Furthermore, nigrostriatal dysfunction can also be detected in clinically unaffected members of kindred with familial PD [120]. In addition, reduction of DAT binding has been reported in subjects with a high genetic risk of PD, suggesting that nigrostriatal dopaminergic dysfunction may be considered a subclinical manifestation of the disease [121,122]. The SN is neither the earliest nor the most severely affected region, and PD-related lesions are seen in the lower brainstem and in olfactory bulb/anterior olfactory nucleus prior to involvement of the SN [123]. Nevertheless, once the involvement of the SN occurs, it still takes time before full parkinsonism can be diagnosed. Indeed, the cardinal motor signs of PD (bradykinesia, rigidity, and resting tremor), which are attributed mostly to dopaminergic cell loss, emerge when there is already a loss of approximately 30% of SN neurons and 50% to 70% of dopaminergic markers in the striatum [124].

Redundancy in basal ganglia functions and mechanisms of functional compensation could serve to delay the clinical onset of PD motor signs during early stages. From this point of view, a late appearance of parkinsonian symptoms would be due to failure of compensatory mechanisms to maintain dopaminergic control over striatal cell function [125]. It is possible that compensatory processes within and outside the basal ganglia, which may be particularly relevant in the early presymptomatic phase of PD, could interfere with the detection of functional impairments for a given period of time [126]. Indeed, regulatory changes that may occur to partially compensate for the loss of dopaminergic terminals include downregulation of the DAT to increase dopaminergic activity and maintain the basal ganglia output within normal limits. Thus, although DAT-SPECT imaging is able to detect reduction of DAT density in patients with PD, we cannot exclude that possible compensatory DAT downregulation at baseline may cause its underestimation and give more severe estimates of DA dysfunction [99]. However, the relevance of DAT downregulation to functional compensation in the preclinical phase is not clear, and it does not appear to reduce sensitivity of the SPECT technique [127].

More recently, adaptive changes in other neural structures than the nigrostriatal pathway, such as the subthalamic nucleus and the globus pallidus pars internalis, have been invoked to play a central role in the compensatory mechanisms that sustain dopaminergic functions in the early phases of PD [128]. It is probable that different mechanisms are likely to come into play at different stages of disease progression [129], and a better recognition of the key mechanisms underlying long-term compensation could lead to the development of therapeutic strategies that aim to further delay the clinical onset of PD [130]. Additionally, these compensatory early events could have long-term, deleterious effects on basal ganglia function and determine the response to drug treatment and the development of motor complications in PD. In particular, it is conceivable that the same mechanisms, which seem to be involved in delaying the onset of clinical symptoms in early disease, may have a different effect in more advanced disease when DA storage capacity and DA availability are further impaired.

Indeed, presynaptic and postsynaptic mechanisms have also been observed to persist in more advanced stages of the disease and have been predicted to promote the development of motor complications as the disease progresses [131]. Specifically, evidence suggests that increased dopamine turnover plays a major role in the pathogenesis of motor fluctuations and dyskinesias [132,133,134]. Functional downregulation of DAT expression has been related to an increased DA turnover and DA release, even accounting for the degree of denervation, contributing to oscillations in synaptic DA levels. Additionally, DA turnover increases after the initiation of levodopa treatment in early PD [135]. This is in keeping with motor complications arising from chronic pulsatile stimulation of DA receptors with levodopa treatment [136]. Such models could explain why patients with dyskinesias tend to express relatively lower levels of DAT per surviving nigrostriatal dopaminergic nerve terminal [137].

Several longitudinal observations indicate that the magnitude of DAT binding in early PD, especially in the posterior putamen, could be predictive for the development of dyskinesias [138] and motor complications in later disease [139]. Of note is that reinstating even a small amount of DAT expression in rats, by grafting cells that express DAT into the striatum of dyskinetic rats, might significantly ameliorate dyskinesias [140]. Furthermore, a recent study demonstrated that the initially low posterior putaminal DAT activity is closely coupled not only with dyskinesia development but also with the timing of levodopa-induced dyskinesia onset in patients with de novo PD [141]. Interestingly, an antero-posterior gradient of dopamine denervation in the putamen, most pronounced in the postero-dorsal area, is maintained throughout the course of Parkinson’s disease, but it is more prominent in early disease [142]. Consistently, baseline DA turnover showed a similar regional pattern with elevated DA turnover in the putamen and further accentuation in its posterior part [139]. In contrast, the lower levels of DAT activity in the anterior putamen of de novo PD patients has been reported to be a significant predictor for future development of motor fluctuations [143]. It remains difficult to explain the underlying pathomechanisms for this regional difference between fluctuations and dyskinesias.

If the loss of DA storage due to presynaptic dopamine neuronal degeneration is preferentially associated with the development of motor fluctuations, dopamine neurons in the anterior putamen could have a greater role in storing DA [143]. However, overall, other functional neuroimaging results have been inconsistent, and the relationship between DAT binding and the emergence of late motor complications of PD remains unclear [144,145]. In particular, postsynaptic changes and the influence of neurotransmitters other than dopamine seem to be involved in their development [146].

Similarly, the relationship between DAT binding and PD progression remains unclear. Bearing in mind that reduced DAT expression might indicate neuronal and axonal dysfunction without alterations in dopaminergic neuronal counts, several authors investigated whether the baseline dopaminergic striatal uptake could predict the development of clinically important long-term motor and nonmotor outcomes. Ravina and colleagues reported that lower [123I]b-CIT SPECT at baseline is independently associated with motor-related disability as well as with nonmotor features such as cognitive impairment, psychosis, and depression [147]. Accordingly, in other individual studies, patients with a more severe reduction of striatal DAT uptake had higher incidence of anxiety [148], visual hallucinations [149], apathy [150], freezing of gait [151], and urinary symptoms [152]. However, imaging markers were not incorporated in the useful predictive model of PD motor progression constructed by Latourelle and colleagues because of their demonstrated scant prognostic utility [153].

The emergence of new DAT tracers promises to result in the development of powerful markers for PD. Furthermore, it is noteworthy to mention that SPECT imaging is limited by poor spatial and temporal resolution and has a worse quantitative capacity than PET. Recently, Li et al. identified ^11^C-PE2, a highly specific DAT radioligand suitable for PET imaging, as a potential alternative surrogate biomarker for studies of PD progression for its ability to track changes in motor performance over time in 33 PD subjects [154]. Overall, findings about the prognostic implications of abnormal DAT imaging in early PD are discordant. Moreover, following PD progression with DAT imaging can be confounded by dopaminergic and other medications. In addition to aging, in terms of clinical confounding factors that determine the initial striatal DAT activity, gender [116] and body mass index [155] have also been suggested to have differential effects in the striatal binding of [123I]FP-CIT.

## 5. Conclusions

DAT Imaging is a reliable tool for investigating the presynaptic dopaminergic nigrostriatal pathway, and it is useful in clinical practice as proof of degenerative parkinsonisms. However, there are several findings that DAT expression is not merely related to the vitality of the nigral neurons, since synapsis involvement could antedate nigral cellular degeneration and DAT density could be modulated as a compensatory mechanism in preclinical/early PD. All of these points, along with several potential external interferences, make DAT imaging a less reliable marker of disease progression.

## References

[1] Dickson D.W. (2018). Neuropathology of Parkinson disease. Parkinsonism Relat. Disord..

[2] Sulzer D. (2007). Multiple hit hypothesis for dopamine neuron loss in Parkinson’s disease. Trends Neurosci..

[3] Uhl G.R. (2003). Dopamine transporter: Basic science and human variation of a key molecule for dopaminergic function, locomotion, and parkinsonism. Mov. Disord..

[4] Sulzer D., Cragg S.J., Rice M.E. (2016). Striatal dopamine neurotransmission: Regulation of release and uptake. Basal Ganglia.

[5] Mulvihill K.G. (2019). Presynaptic regulation of dopamine release: Role of the DAT and VMAT2 transporters. Neurochem. Int..

[6] Benamer H.T.S., Patterson J., Wyper D.J., Hadley D.L., Macphee G.J.A., Grosset D.G. (2000). Correlation of Parkinsons disease severity and duration with [123I]FP-CIT SPECT striatal uptake. Mov. Disord..

[7] Spiegel J., Hellwig D., Samnick S., Jost W., Möllers M.O., Fassbender K., Kirsch C.M., Dillmann U. (2007). Striatal FP-CIT uptake differs in the subtypes of early Parkinson’s disease. J. Neural Transm..

[8] Rossi C., Frosini D., Volterrani D., De Feo P., Unti E., Nicoletti V., Kiferle L., Bonuccelli U., Ceravolo R. (2010). Differences in nigro-striatal impairment in clinical variants of early Parkinson’s disease: Evidence from a FP-CIT SPECT study. Eur. J. Neurol..

[9] Booij T., Tissingh G., Boer G., Speelman J.D., Stoof J.C., Janssen A.G., Wolters E.C., van Royen E.A. (1997). [123I]FP-SPECT shows a pronounced decline of striatal dopamine transporter labelling in early and advanced Parkinson’s disease. J. Neurol. Neurosurg. Psychiatry.

[10] Mozley P.D., Schneider J.S., Acton P.D., Plössl K., Stern M.B., Siderowf A., Leopold N.A., Li P.Y., Alavi A., Kung H.F. (2000). Binding of [99mTc- ]TRODAT-1 to dopamine transporters in patients with Parkinson’s disease and in healthy volunteers. J. Nucl. Med..

[11] Marek K., Innis R.B., Van Dyck C.H., Fussell B., Early M., Eberly S., Oakes D., Seibyl J. (2001). [123I]beta-CIT SPECT imaging assessment of the rate of Parkinson’s disease progression. Neurology.

[12] Kim H.J., Im J.H., Yang S.O., Moon D.H., Ryu J.S., Bong J.K., Nam K.P., Cheon J.H., Lee M.C., Lee H.K. (1997). Imaging and quantitation of dopamine transporters with iodine-123-IPT in normal and Parkinson’s disease subjects. J. Nucl. Med..

[13] Tatsch K., Poepperl G. (2013). Nigrostriatal dopamine terminal imaging with dopamine transporter SPECT: An update. J. Nucl. Med..

[14] Saeed U., Compagnone J., Aviv R.I., Strafella A.P., Black S.E., Lang A.E., Masellis M. (2017). Imaging biomarkers in Parkinson’s disease and Parkinsonian syndromes: Current and emerging concepts. Transl. Neurodegener..

[15] Bajaj N., Hauser R.A., Grachev I.D. (2013). Clinical utility of dopamine transporter single photon emission CT (DaT-SPECT) with (123I) ioflupane in diagnosis of parkinsonian syndromes. J. Neurol. Neurosurg. Psychiatry.

[16] Gerschlager W., Bencsits G., Pirker W., Bloem B.R., Asenbaum S., Prayer D., Zijlmans J.C., Hoffmann M., Brücke T. (2002). [123I]beta-CIT SPECT distinguishes vascular parkinsonism from Parkinson’s disease. Mov. Disord..

[17] Lorberboym M., Djaldetti R., Melamed E., Sadeh M., Lampl Y. (2004). 123I-FP-CIT SPECT imaging of dopamine transporters in patients with cerebrovascular disease and clinical diagnosis of vascular parkinsonism. J. Nucl. Med..

[18] Antonini A., Vitale C., Barone P., Cilia R., Righini A., Bonuccelli U., Abbruzzese G., Ramat S., Petrone A., Quatrale R. (2012). The relationship between cerebral vascular disease and parkinsonism: The VADO study. Parkinsonism Relat. Disord..

[19] Benítez-Rivero S., Marín-Oyaga V.A., García-Solís D., Huertas-Fernández I., García-Gómez F.J., Jesús S., Cáceres M.T., Carrillo F., Ortiz A.M., Carballo M. (2013). Clinical features and 123I-FP-CIT SPECT imaging in vascular parkinsonism and Parkinson’s disease. J. Neurol. Neurosurg. Psychiatry.

[20] Tolosa E., Coelho M., Gallardo M. (2003). DAT imaging in drug-induced and psychogenic parkinsonism. Mov. Disord..

[21] Lorberboym M., Treves T.A., Melamed E., Lampl Y., Hellmann M., Djaldetti E. (2006). [123I]-FP/CIT SPECT imaging for distinguishing drug-induced parkinsonism from Parkinson’s disease. Mov. Disord..

[22] Tinazzi M., Cipriani A., Matinella A., Cannas A., Solla P., Nicoletti A., Zappia M., Morgante L., Morgante F., Pacchetti C. (2012). [123I]FP-CIT single photon emission computed tomography findings in drug-induced Parkinsonism. Schizophr. Res..

[23] Ceravolo R., Antonini A., Volterrani D., Rossi C., Kiferle L., Frosini D., Lucetti C., Isaias I.U., Benti R., Murri L. (2008). Predictive value of nigrostriatal dysfunction in isolated tremor: A clinical and SPECT study. Mov. Disord..

[24] Novellino F., Arabia G., Bagnato A., Cascini G.L., Salsone M., Nicoletti G., Messina D., Morelli M., Paglionico S., Giofrè L. (2009). Combined use of DAT-SPECT and cardiac MIBG scintigraphy in mixed tremors. Mov. Disord..

[25] Arabia G., Novellino F., Morelli M., Paglionico S., Salsone M., Giofrè L., Pucci F., Bagnato A., Cascini G.L., Nicoletti G. (2010). Mixed tremors with integrity of nigrostriatal system: A clinical and DAT-SPECT follow-up study. Mov. Disord..

[26] De Verdal M., Renard D., Collombier L., Boudousq V., Kotzki P.O., Labauge P., Castelnovo G. (2013). I123-FP-CIT single-photon emission tomography in patients with long-standing mixed tremor. Eur. J. Neurol..

[27] Postuma R.B., Berg D., Stern M., Poewe W., Olanow C.W., Oertel W., Obeso J., Marek K., Litvan I., Lang A.E. (2015). MDS clinical diagnostic criteria for Parkinson’s disease. Mov. Disord..

[28] Erro R., Schneider S.A., Stamelou M., Quinn N.P., Bhatia K.P. (2016). What do patients with scans without evidence of dopaminergic deficit (SWEDD) have? New evidence and continuing controversies. J. Neurol. Neurosurg. Psychiatry.

[29] Sixel-Döring F., Liepe K., Mollenhauer B., Trautmann E., Trenkwalder C. (2011). The role of 123I-FP-CIT-SPECT in the differential diagnosis of Parkinson and tremor syndromes: A critical assessment of 125 cases. J. Neurol..

[30] Marshall V.L., Patterson J., Hadley D.M., Grosset K.A., Grosset D.G. (2006). Two-year follow-up in 150 consecutive cases with normal dopamine transporter imaging. Nucl. Med. Commun..

[31] Wyman-Chick K.A., Martin P.K., Minar M., Schroeder R.W. (2016). Cognition in patients with a clinical diagnosis of Parkinson disease and scans without evidence of dopaminergic deficit (SWEDD): 2-year follow-up. Cogn. Behav. Neurol..

[32] Mille E., Levin J., Brendel M., Zach C., Barthel H., Sabri O., Bötzel K., Bartenstein P., Danek A., Rominger A. (2017). Cerebral glucose metabolism and dopaminergic function in patients with corticobasal syndrome. J. Neuroimaging.

[33] McKeith I., O’Brien J., Walker Z., Tatsch K., Booij J., Darcourt J., Padovani A., Giubbini R., Bonuccelli U., Volterrani D. (2007). Sensitivity and specificity of dopamine transporter imaging with 123I-FP-CIT SPECT in dementia with Lewy bodies: A phase III, multicentre study. Lancet Neurol..

[34] Thomas A.J., Attems J., Colloby S.J., O’Brien J.T., McKeith I., Walker R., Lee L., Burn D., Lett D.J., Walker Z. (2017). Autopsy validation of 123I-FP-CIT dopaminergic neuroimaging for the diagnosis of DLB. Neurology.

[35] Cilia R., Rossi C., Frosini D., Volterrani D., Siri C., Pagni C., Benti R., Pezzoli G., Bonuccelli U., Antonini A. (2011). Dopamine transporter SPECT imaging in corticobasal syndrome. PLos ONE.

[36] Ceravolo R., Rossi C., Cilia R., Tognoni G., Antonini A., Volterrani D., Bonuccelli U. (2013). Evidence of delayed nigrostriatal dysfunction in corticobasal syndrome: A SPECT follow-up study. Parkinsonism Relat. Disord..

[37] Pirker S., Perju-Dumbrava L., Kovacs G.G., Traub-Weidinger T., Pirker W. (2015). Progressive dopamine transporter binding loss in autopsy-confirmed corticobasal degeneration. J. Parkinsons Dis..

[38] Van der Zande J.J., Booij J., Scheltens P., Raijmakers P.G., Lemstra A.W. (2016). [(123)]FP-CIT SPECT scans initially rated as normal became abnormal over time in patients with probable dementia with Lewy bodies. Eur. J. Nucl. Med. Mol. Imaging.

[39] Filippi L., Manni C., Pierantozzi M., Brusa L., Danieli R., Stanzione P., Schillaci O. (2006). 123I-FP-CIT in progressive supranuclear palsy and in Parkinson’s disease: A SPECTsemiquantitative study. Nucl. Med. Commun..

[40] Perju-Dumbrava L.D., Kovacs G.G., Pirker S., Jellinger K., Hoffmann M., Asenbaum S., Pirker W. (2012). Dopamine transporter imaging in autopsy-confirmed Parkinson’s disease and multiple system atrophy. Mov. Disord..

[41] Matesan M., Gaddikeri S., Longfellow K., Miyaoka R., Elojeimy S., Elman S., Hu S.C., Minoshima S., Lewis D. (2018). I-123 DaTscan SPECT Brain Imaging in Parkinsonian Syndromes: Utility of the Putamen-to-Caudate Ratio. J. Neuroimaging.

[42] Kahraman D., Eggers C., Schicha H., Timmermann L., Schmidt M. (2012). Visual assessment of dopaminergic degeneration pattern in 123I-FP-CIT SPECT differentiates patients with atypical parkinsonian syndromes and idiopathic Parkinson’s disease. J. Neurol..

[43] Davidsson A., Georgiopoulos C., Dizdar N., Granerus G., Zachrisson H. (2014). Comparison between visual assessment of dopaminergic degeneration pattern and semi-quantitative ratio calculations in patients with Parkinson’s disease and atypical parkinsonian syndromes using DaTSCAN (R) SPECT. Ann. Nucl. Med..

[44] Brooks D.J. (2016). Imaging of genetic and degenerative disorders primarily causing Parkinsonism. Handb. Clin. Neurol..

[45] Torres G.E. (2006). The dopamine transporter proteome. J. Neurochem..

[46] Ikawa K., Watanabe A., Kaneno S., Toru M. (1993). Modulation of [3H]mazindol binding sites in rat striatum by dopaminergic agents. Eur. J. Pharmacol..

[47] Rioux L., Frohna P.A., Joyce J.N., Schneider J.S. (1997). The effects of chronic levodopa treatment on pre- and postsynaptic markers of dopaminergic function in striatum of parkinsonian monkeys. Mov. Disord..

[48] Murer M.G., Dziewczapolski G., Menalled L.B., García M.C., Agid Y., Gershanik O., Raisman-Vozari R. (1998). Chronic levodopa is not toxic for remaining dopamine neurons, but instead promotes their recovery, in rats with moderate nigrostriatal lesions. Ann. Neurol..

[49] Gnanalingham K.K., Robertson R.G. (1994). The effects of chronic continuous versus intermittent levodopa treatments on striatal and extrastriatal D1 and D2 dopamine receptors and dopamine uptake sites in the 6-hydroxydopamine lesioned rat–an autoradiographic study. Brain Res..

[50] Innis R.B., Marek K.L., Sheff K., Zoghbi S., Castronuovo J., Feigin A., Seibyl J.P. (1999). Effect of treatment with Levodopa-Carbidopa or L-Selegiline on striatal dopamine transporter SPECT imaging with I [123]Beta-CIT. Mov. Disord..

[51] Guttman M., Stewart D., Hussey D., Wilson A., Houle S., Kish S. (2001). Influence of L-Dopa and pramipexole on striatal dopamine transporter in early PD. Neurology.

[52] Ahlskog J.E., Uitti R.J., O’Connor M.K., Maraganore D.M., Matsumoto J.Y., Stark K.F., Turk M.F., Burnett O.L. (1999). The effect of dopamine agonist therapy on dopamine transporter imaging in Parkinson’s disease. Mov. Disord..

[53] Parkinson Study Group (2002). Dopamine transporter brain imaging to assess the effects of pramipexole vs. levodopa on Parkinson disease progression. JAMA.

[54] Bolan E.A., Kivell B., Jaligam V., Oz M., Jayanthi L.D., Han Y., Sen N., Urizar E., Gomes I., Devi L.A. (2007). D2 receptors regulate dopamine transporter function via an extracellular signal-regulated kinases 1 and 2-dependent and phosphoinositide 3 kinase-independent mechanism. Mol. Pharmacol..

[55] Castro-Hernandez J., Afonso-Oramas D., Cruz-Munos I., Salas-Hernández J., Barroso-Chinea P., Moratalla R., Millan M.J., González-Hernández T. (2015). Prolonged treatment with pramipexole promotes physical interaction of striatal dopamine D3 autoreceptors with dopamine transporters to reduce dopamine uptake. Neurobiol. Dis..

[56] Stouffer M.A., Ali S., Reith M.E.A., Patel J.C., Sarti F., Carr K.D., Rice M.E. (2011). SKF-83566, a D1 dopamine receptor antagonist, inhibits the dopamine transporter. J. Neurochem..

[57] Rossi C., Genovesi D., Marzullo P., Giorgetti A., Filidei E., Corsini G.U., Bonuccelli U., Ceravolo R. (2017). Striatal dopamine transporter modulation after rotigotine: Results from a pilot single-photon emission computed tomography study in a group of early stage Parkinson disease patients. Clin. Neuropharmacol..

[58] Ikeda K., Ebina J., Kawabe K., Iwasaki Y. (2019). Dopamine Transporter Imaging in Parkinson Disease: Progressive Changes and Therapeutic Modification after Anti-parkinsonian Medications. Intern. Med..

[59] Kugaya A., Seneca N.M., Snyder P.J., Williams S.A., Malison R.T., Baldwin R.M., Seibyl J.P., Innis R.B. (2003). Changes in human in vivo serotonin and dopamine transporter availabilities during chronic antidepressant administration. Neuropsychopharmacology.

[60] Wu C.K., Chin Chen K., See Chen P., Chiu N.T., Yeh T.L., Lee I.H., Yang Y.K. (2013). No changes in striatal dopamine transporter in antidepressant-treated patients with major depression. Int. Clin. Psychopharmacol..

[61] Taylor J.P., Colloby S.J., McKeith I.G., Burn D.J., Williams D., Patterson J., O’Brien J.T. (2007). Cholinesterase inhibitor use does not significantly influence the ability of 123I-FP-CIT imaging to distinguish Alzheimer’s disease from dementia with Lewy bodies. J. Neurol. Neurosurg. Psychiatry.

[62] Gigante A.F., Asabella A.N., Iliceto G., Martino T., Ferrari C., Defazio G., Rubini G. (2018). Chronic coffee consumption and striatal DAT-SPECT findings in Parkinson’s disease. Neurol. Sci..

[63] Yang Y.K., Yao W.J., Yeh T.L., Lee I.H., Chen P.S., Lu R.B., Chiu N.T. (2008). Decreased dopamine transporter availability in male smokers—A dual isotope SPECT study. Prog. Neuropsychopharmacol. Biol. Psychiatry.

[64] Ashok A.H., Mizuno Y., Howes O.D. (2019). Tobacco smoking and dopaminergic function in humans: A meta-analysis of molecular imaging studies. Psychopharmacology.

[65] Proebstl L., Kamp F., Manz K., Krause D., Adorjan K., Pogarell O., Koller G., Soyka M., Falkai P., Kambeitz J. (2019). Effects of stimulant drug use on the dopaminergic system: A systematic review and meta-analysis of in vivo neuroimaging studies. Eur. Psychiatry.

[66] Bezard E., Dovero S., Prunier C., Ravenscroft P., Chalon S., Guilloteau D., Crossman A.R., Bioulac B., Brotchie J.M., Gross C.E. (2001). Relationship between the appearance of symptoms and the level of nigrostriatal degeneration in a progressive 1-methyl-4-phenyl-1,2,3,6 tetrahydropyridinelesioned macaque model of Parkinson’s disease. J. Neurosci..

[67] Colloby S., McParland S., O’Brien J.T., Attems J. (2012). Neuropathological correlates of dopaminergic imaging in Alzheimer’s disease and Lewy body dementias. Brain.

[68] Kraemmer J., Kovacs G.G., Perju-Dumbrava L., Pirker S., Traub-Weidinger T., Pirker W. (2014). Correlation of striatal dopamine transporter imaging with post mortem substantia nigra cell counts. Mov. Disord..

[69] Karimi M., Tian L., Brown C.A., Flores H.P., Loftin S.K., Videen T.O., Moerlein S.M., Perlmutter J.S. (2013). Validation of nigrostriatal positron emission tomography measures: Critical limits. Ann. Neurol..

[70] Saari L., Kivinen K., Gardberg M., Joutsa J., Noponen T., Kaasinen V. (2017). Dopamine transporter imaging does not predict the number of nigral neurons in Parkinson disease. Neurology.

[71] Sasaki M., Shibata E., Tohyama K., Takahashi J., Otsuka K., Tsuchiya K., Takahashi S., Ehara S., Terayama Y., Sakai A. (2006). Neuromelanin magnetic resonance imaging of locus ceruleus and substantia nigra in Parkinson’s disease. Neuroreport.

[72] Ohtsuka C., Sasaki M., Konno K., Koide M., Kato K., Takahashi J., Takahashi S., Kudo K., Yamashita F., Terayama Y. (2013). Changes in substantia nigra and locus coeruleus in patients with early-stage Parkinson’s disease using neuromelanin-sensitive MR imaging. Neurosci. Lett..

[73] Ogisu K., Kudo K., Sasaki M., Sakushima K., Yabe I., Sasaki H., Terae S., Nakanishi M., Shirato H. (2013). 3D neuromelanin-sensitive magnetic resonance imaging with semi-automated volume measurement of the sub stantia nigra pars compacta for diagnosis of Parkinson’s disease. Neuroradiology.

[74] Ohtsuka C., Sasaki M., Konno K., Kato K., Takahashi J., Yamashita F., Terayama Y. (2014). Differentiation of early-stage parkinsonisms using neuromelanin-sensitive magnetic resonance imaging. Parkinsonism Relat. Disord..

[75] Reimao S., Pita Lobo P., Neutel D., Correia Guedes L., Coelho M., Rosa M.M., Ferreira J., Abreu D., Gonçalves N., Morgado C. (2015). Substantia nigra neuromelanin magnetic resonance imaging in de novo Parkinson’s disease patients. Eur. J. Neurol..

[76] Schwarz S.T., Xing Y., Tomar P., Bajaj N., Auer D.P. (2017). In vivo assessment of brainstem depigmentation in Parkinson disease: Potential as a severity marker for multicenter studies. Radiology.

[77] Prasad S., Stezin A., Lenka A., George L., Saini J., Yadav R., Pal P.K. (2018). Three-dimensional neuromelanin-sensitive magnetic resonance imaging of the substantia nigra in Parkinson’s disease. Eur. J. Neurol..

[78] Pavese N., Tai Y.F. (2018). Nigrosome Imaging and Neuromelanin Sensitive MRI in Diagnostic Evaluation of Parkinsonism. Mov. Disord. Clin. Pract..

[79] Reimao S., Pita Lobo P., Neutel D., Guedes L.C., Coelho M., Rosa M.M., Azevedo P., Ferreira J., Abreu D., Gonçalves N. (2015). Substantia nigra neuromelanin-MR imaging differentiates essential tremor from Parkinson’s disease. Mov. Disord..

[80] Kashihara K., Shinya T., Higaki F. (2011). Neuromelanin magnetic resonance imaging of nigral volume loss in patients with Parkinson’s disease. J. Clin. Neurosci..

[81] Miyoshi F., Ogawa T., Kitao S.I., Kitayama M., Shinohara Y., Takasugi M., Fujii S., Kaminou T. (2013). Evaluation of Parkinson disease and Alzheimer disease with the use of neuromelanin MR imaging and (123)Imetaiodobenzylguanidine scintigraphy. AJNR Am. J. Neuroradiol..

[82] Kitao S., Matsusue E., Fujii S., Miyoshi F., Kaminou T., Kato S., Ito H., Ogawa T. (2013). Correlation between pathology and neuromelanin MR imaging in Parkinson’s disease and dementia with Lewy bodies. Neuroradiology.

[83] Martín-Bastida A., Lao-Kaim N.P., Roussakis A.A., Searle G.E., Xing Y., Gunn R.N., Schwarz S.T., Barker R.A., Auer D.P., Piccini P. (2019). Relationship between neuromelanin and dopamine terminals within the Parkinson’s nigrostriatal system. Brain.

[84] Kuya K., Shinohara Y., Miyoshi F., Fujii S., Tanabe Y., Ogawa T. (2016). Correlation between neuromelanin-sensitive MR imaging and (123)I-FP-CIT SPECT in patients with parkinsonism. Neuroradiology.

[85] Isaias I.U., Trujillo P., Summers P., Marotta G., Mainardi L., Pezzoli G., Zecca L., Costa A. (2016). Neuromelanin imaging and dopaminergic loss in Parkinson’s disease. Front. Aging Neurosci..

[86] Lehericy S., Sharman M.A., Dos Santos C.L., Paquin R., Gallea C. (2012). Magnetic resonance imaging of the substantia nigra in Parkinson’s disease. Mov. Disord..

[87] Cosottini M., Frosini D., Pesaresi I., Costagli M., Biagi L., Ceravolo R., Bonuccelli U., Tosetti M. (2014). MR imaging of the substantia nigra at 7 T enables diagnosis of Parkinson disease. Radiology.

[88] Blazejewska A.I., Schwarz S.T., Pitiot A., Stephenson M.C., Lowe J., Bajaj N., Bowtell R.W., Auer D.P., Gowland P.A. (2013). Visualization of nigrosome 1 and its loss in PD: Pathoanatomical correlation and in vivo 7 T MRI. Neurology.

[89] Lehericy S., Vaillancourt D.E., Seppi K., Monchi O., Rektorova I., Antonini A., McKeown M.J., Masellis M., Berg D., Rowe J.B. (2017). The role of high-field magnetic resonance imaging in parkinsonian disorders: Pushing the boundaries forward. Mov. Disord..

[90] Postuma R.B., Berg D. (2016). Advances in markers of prodromal Parkinson disease. Nat. Rev. Neurol..

[91] Iranzo A., Lomeña F., Stockner H., Valldeoriola F., Vilaseca I., Salamero M., Molinuevo J.L., Serradell M., Duch J., Pavía J. (2010). Decreased striatal dopamine transporter uptake and substantia nigra hyperechogenicity as risk markers of synucleinopathy in patients with idiopathic rapid-eye-movement sleep behaviour disorder: A prospective study. Lancet Neurol..

[92] Li Y., Kang W., Yang Q., Zhang L., Zhang L., Dong F., Chen S., Liu J. (2017). Predictive markers for early conversion of iRBD to neurodegenerative synucleinopathy diseases. Neurology.

[93] Iranzo A., Santamaria J., Valldeoriola F., Serradell M., Salamero M., Gaig C., Niñerola-Baizán A., Sánchez-Valle R., Lladó A., De Marzi R. (2017). Dopamine transporter imaging deficit predicts early transition to synucleinopathy in idiopathic REM sleep behavior disorder. Ann. Neurol..

[94] Ceravolo R., Antonini A., Frosini D., De Iuliis A., Weis L., Cecchin D., Tosetti M., Bonuccelli U., Cosottini M. (2015). Nigral anatomy and striatal denervation in genetic parkinsonism: A family report. Mov. Disord..

[95] De Marzi R., Seppi K., Hogl B., Müller C., Scherfler C., Stefani A., Iranzo A., Tolosa E., Santamarìa J., Gizewski E. (2016). Loss of dorsolateral nigral hyperintensity on 3.0 tesla susceptibility-weighted imaging in idiopathic rapid eye movement sleep behavior disorder. Ann. Neurol..

[96] Bae Y.J., Kim J.M., Kim E., Lee K.M., Kang S.Y., Park H.S., Kim K.J., Kim Y.E., Oh E.S., Yun J.Y. (2016). Loss of nigral hyperintensity on 3 Tesla MRI of parkinsonism: Comparison with (123) I-FP-CIT SPECT. Mov. Disord..

[97] Frosini D., Ceravolo R., Tosetti M., Bonuccelli U., Cosottini M. (2016). Nigral involvement in atypical parkinsonisms: Evidence from a pilot study with ultra-high field MRI. J. Neural Transm..

[98] Frosini D., Cosottini M., Donatelli G., Costagli M., Biagi L., Pacchetti C., Terzaghi M., Cortelli P., Arnaldi D., Bonanni E. (2017). Seven tesla MRI of the substantia nigra in patients with rapid eye movement sleep behavior disorder. Parkinsonism Relat. Disord..

[99] Lee C.S., Samii A., Sossi V., Ruth T.J., Schulzer M., Holden J.E., Wudel J., Pal P.K., de la Fuente-Fernandez R., Calne D.B. (2000). In vivo positron emission tomographic evidence for compensatory changes in presynaptic dopaminergic nerve terminals in Parkinson’s disease. Ann. Neurol..

[100] Schirinzi T., Madeo G., Martella G., Maltese M., Picconi B., Calabresi P., Pisani A. (2016). Early synaptic dysfunction in Parkinson’s disease: Insights from animal models. Mov. Disord..

[101] Cheng H.C., Ulane C.M., Burke R.E. (2010). Clinical progression in Parkinson disease and the neurobiology of axons. Ann. Neurol..

[102] Dijkstra A.A., Ingrassia A., de Menezes R.X., van Kesteren R.E., Rozemuller A.J., Heutink P., van de Berg W.D. (2015). Evidence for Immune Response, Axonal Dysfunction and Reduced Endocytosis in the Substantia Nigra in Early Stage Parkinson’s Disease. PLos ONE.

[103] Hornykiewicz O. (1998). Biochemical aspects of Parkinson’s disease. Neurology.

[104] Sian-Hulsmann J., Monoranu C., Strobel S., Riederer P. (2015). Lewy bodies: A spectator or salient killer?. Cns Neurol. Disord. Drug Targets.

[105] Simuni T., Siderowf A., Lasch S., Coffey C.S., Caspell-Garcia C., Jennings D., Tanner C.M., Trojanowski J.Q., Shaw L.M., Seibyl J. (2018). Parkinson’s Progression Marker Initiative. Longitudinal change of clinical and biological measures in early Parkinson’s disease: Parkinson’s Progression Markers Initiative cohort. Mov. Disord..

[106] Kordower J.H., Olanow C.W., Dodiya H.B., Chu Y., Beach T.G., Adler C.H., Halliday G.M., Bartus R.T. (2013). Disease duration and the integrity of the nigrostriatal system in Parkinson’s disease. Brain.

[107] Bridi J.C., Hirth F. (2018). Mechanisms of α-Synuclein Induced Synaptopathy in Parkinson’s Disease. Front. Neurosci..

[108] Phan J.A., Stokholm K., Zareba-Paslawska J., Jakobsen S., Vang K., Gjedde A., Landau A.M., Romero-Ramos M. (2017). Early synaptic dysfunction induced by α-synuclein in a rat model of Parkinson’s disease. Sci. Rep..

[109] Ross G.W., Petrovitch H., Abbott R.D., Nelson J., Markesbery W., Davis D., Hardman J., Launer L., Masaki K., Tanner C.M. (2004). Parkinsonian signs and substantia nigra neuron density in decendents elders without PD. Ann. Neurol..

[110] Dorsey E.R., Sherer T., Okun M.S., Bloem B.R. (2018). The Emerging Evidence of the Parkinson Pandemic. J. Parkinsons Dis..

[111] Tysnes O.B., Storstein A. (2017). Epidemiology of Parkinson’s disease. J. Neural Transm..

[112] Kempster P.A., O’Sullivan S.S., Holton J.L., Revesz T., Lees A.J. (2010). Relationships between age and late progression of Parkinson’s disease: A clinico-pathological study. Brain.

[113] Kish S.J., Shannak K., Rajput A., Deck J.H., Hornykiewicz O. (1992). Aging produces a specific pattern of striatal dopamine loss: Implications for the etiology of idiopathic Parkinson’s disease. J. Neurochem..

[114] Cruz-Muros I., Afonso-Oramas D., Abreu P., Pérez-Delgado M.M., Rodríguez M., González-Hernández T. (2009). Aging effects on the dopamine transporter expression and compensatory mechanisms. Neurobiol. Aging.

[115] Lee C.S., Kim S.J., Oh S.J., Kim H.O., Yun S.C., Doudet D., Kim J.S. (2014). Uneven age effects of [(18)F]FP-CIT binding in the striatum of Parkinson’s disease. Ann. Nucl. Med..

[116] Kaasinen V., Joutsa J., Noponen T., Johansson J., Seppänen M. (2015). Effects of aging and gender on striatal and extrastriatal [123I]FP-CIT binding in Parkinson’s disease. Neurobiol. Aging.

[117] Darbin O. (2012). The aging striatal dopamine function. Parkinsonism Relat. Disord..

[118] Iacono D., Geraci-Erck M., Rabin M.L., Adler C.H., Serrano G., Beach T.G., Kurlan R. (2015). Parkinson disease and incidental Lewy body disease: Just a question of time?. Neurology.

[119] Gaig C., Tolosa E. (2009). When does Parkinson’s disease begin?. Mov. Disord..

[120] Piccini P., Morrish P.K., Turjanski N., Sawle G.V., Burn D.J., Weeks R.A., Mark M.H., Maraganore D.M., Lees A.J., Brooks D.J. (1997). Dopaminergic function in familial Parkinson’s disease: A clinical and [18F]dopa positron emission tomography study. Ann. Neurol..

[121] Piccini P., Burn D.J., Ceravolo R., Maraganore D., Brooks D.J. (1999). The role of inheritance in sporadic Parkinson’s disease: Evidence from a longitudinal study of dopaminergic function in twins. Ann. Neurol..

[122] Adams J.R., van Netten H., Schulzer M., Mak E., Mckenzie J., Strongosky A., Sossi V., Ruth T.J., Lee C.S., Farrer M. (2005). PET inLRRK2 mutations: Comparison to sporadic Parkinson’s disease and evidence for presymptomatic compensation. Brain.

[123] Beach T.G., Adler C.H., Lue L., Sue L.I., Bachalakuri J., Henry-Watson J., Sasse J., Boyer S., Shirohi S., Brooks R. (2009). Unified staging system for Lewy body disorders: Correlation with nigrostriatal degeneration, cognitive impairment and motor dysfunction. Acta Neuropathol..

[124] Greffard S., Verny M., Bonnet A.M., Beinis J.Y., Gallinari C., Meaume S., Piette F., Hauw J.J., Duyckaerts C. (2006). Motor score of the Unified Parkinson Disease Rating Scale as a good predictor of Lewy body-associated neuronal loss in the substantia nigra. Arch. Neurol..

[125] Zigmond M.J., Abercrombie E.D., Berger T.W., Grace A.A., Stricker E.M. (1990). Compensations after lesions of central dopaminergic neurons: Some clinical and basic implications. Trends Neurosci..

[126] Obeso J.A., Rodriguez-Oroz M.C., Lanciego J.L., Diaz M.R., Rodriguez Diaz M. (2004). How does Parkinson’s disease begin? The role of compensatory mechanisms. Trends Neurosci..

[127] Kagi G., Bhatia K.P., Tolosa E. (2010). The role of DAT-SPECT in movement disorders. J. Neurol. Neurosurg. Psychiatry.

[128] Bezard E., Gross C.E., Brotchie J.M. (2003). Presymptomatic compensation in Parkinson’s disease is not dopamine-mediated. Trends Neurosci..

[129] Brotchie J., Fitzer-Attas C. (2009). Mechanisms compensating for dopamine loss in early Parkinson disease. Neurology.

[130] Blesa J., Trigo-Damas I., Dileone M., Del Rey N.L., Hernandez L.F., Obeso J.A. (2017). Compensatory mechanisms in Parkinson’s disease: Circuits adaptations and role in disease modification. Exp. Neurol..

[131] De la Fuente-Fernández R., Pal P.K., Vingerhoets F.J., Kishore A., Schulzer M., Mak E.K., Ruth T.J., Snow B.J., Calne D.B., Stoessl A.J. (2000). Evidence for impaired presynaptic dopamine function in parkinsonian patients with motor fluctuations. J. Neural Transm..

[132] De la Fuente-Fernàndez R., Lu J.Q., Sossi V., Jivan S., Schulzer M., Holden J.E., Lee C.S., Ruth T.J., Calne D.B., Stoessl A.J. (2001). Biochemical variations in the synaptic level of dopamine precede motor fluctuations in Parkinson’s disease: PET evidence of increased dopamine turnover. Ann. Neurol..

[133] De la Fuente-Fernàndez R., Sossi V., Huang Z., Furtado S., Lu J.Q., Calne D.B., Ruth T.J., Stoessl A.J. (2004). Levodopa induced changes in synaptic dopamine levels increase with progression of Parkinson’s disease: Implications for dyskinesias. Brain.

[134] De la Fuente-Fernàndez R., Schulzer M., Mak E., Calne D.B., Stoessl A.J. (2004). Presynaptic mechanisms of motor fluctuations in Parkinson’s disease: A probabilistic model. Brain.

[135] Sossi V., de la Fuente-Fernández R., Schulzer M., Troiano A.R., Ruth T.J., Stoessl A.J. (2007). Dopamine transporter relation to dopamine turnover in Parkinson’s disease: A positron emission tomography study. Ann. Neurol..

[136] Stoessl A.J. (2015). Central pharmacokinetics of levodopa: Lessons from imaging studies. Mov. Disord..

[137] Troiano A.R., de la Fuente-Fernàndez R., Sossi V., Schulzer M., Mak E., Ruth T.J., Stoessl A.J. (2009). PET demonstrates reduced dopamine transporter expression in PD with dyskinesias. Neurology.

[138] Hong J.Y., Oh J.S., Lee I., Sunwoo M.K., Ham J.H., Lee J.E., Sohn Y.H., Kim J.S., Lee P.H. (2014). Presynaptic dopamine depletion predicts levodopa-induced dyskinesia in de novo Parkinson disease. Neurology.

[139] Löhle M., Mende J., Wolz M., Beuthien-Baumann B., Oehme L., van den Hoff J., Kotzerke J., Reichmann H., Storch A. (2016). Putaminal dopamine turnover in de novo Parkinson disease predicts later motor complications. Neurology.

[140] Tomas D., Stanic D., Chua H.K., White K., Boon W.C., Horne M. (2016). Restoration of the Dopamine Transporter through Cell Therapy Improves Dyskinesia in a Rat Model of Parkinson’s Disease. PLos ONE.

[141] Yoo H.S., Chung S.J., Chung S.J., Moon H., Oh J.S., Kim J.S., Hong J.Y., Ye B.S., Sohn Y.H., Lee P.H. (2018). Presynaptic dopamine depletion determines the timing of levodopa-induced dyskinesia onset in Parkinson’s disease. Eur. J. Nucl. Med. Mol. Imaging.

[142] Nandhagopal R., Kuramoto L., Schulzer M., Mak E., Cragg J., Lee C.S., McKenzie J., McCormick S., Samii A., Troiano A. (2009). Longitudinal progression of sporadic Parkinson’s disease: A multitracer positron emission tomography study. Brain.

[143] Chung S.J., Lee Y., Oh J.S., Kim J.S., Lee P.H., Sohn Y.H. (2018). Putaminal dopamine depletion in de novo Parkinson’s disease predicts future development of wearing-off. Parkinsonism Relatdisord..

[144] Linazasoro G., Van Blercom N., Bergaretxe A., Iñaki F.M., Laborda E., Ruiz Ortega J.A. (2009). Levodopa-induced dyskinesias in Parkinson disease are independent of the extent of striatal dopaminergic denervation: A pharmacological and SPECT study. Clin. Neuropharmacol..

[145] Djaldetti R., Rigbi A., Greenbaum L., Reiner J., Lorberboym M. (2018). Can early dopamine transporter imaging serve as a predictor of Parkinson’s disease progression and late motor complications?. J. Neurol. Sci..

[146] Calabresi P., Di Filippo M., Ghiglieri V., Tambasco N., Picconi B. (2010). Levodopa-induced dyskinesias in patients with Parkinson’s disease: Filling the bench-to-bedside gap. Lancet Neurol..

[147] Ravina B., Marek K., Eberly S., Oakes D., Kurlan R., Ascherio A., Beal F., Beck J., Flagg E., Galpern W.R. (2012). Dopamine transporter imaging is associated with long-term outcomes in Parkinson’s disease. Mov. Disord..

[148] Erro R., Pappatà S., Amboni M., Vicidomini C., Longo K., Santangelo G., Picillo M., Vitale C., Moccia M., Giordano F. (2012). Anxiety is associated with striatal dopamine transporter availability in newly diagnosed untreated Parkinson’s disease patients. Parkinsonism Relat. Disord..

[149] Kiferle L., Ceravolo R., Giuntini M., Linsalata G., Puccini G., Volterrani D., Bonuccelli U. (2014). Caudate dopaminergic denervation and visual hallucinations: Evidence from a 123I-FP-CIT SPECT study. Parkinsonism Relat. Disord..

[150] Santangelo G., Vitale C., Picillo M., Cuoco S., Moccia M., Pezzella D., Erro R., Longo K., Vicidomini C., Pellecchia M.T. (2015). Apathy and striatal dopamine transporter levels in de-novo, untreated Parkinson’s disease patients. Parkinsonism Relat. Disord..

[151] Kim R., Lee J., Kim Y., Kim A., Jang M., Kim H.J., Jeon B., Kang U.J., Fahn S. (2018). Presynaptic striatal dopaminergic depletion predicts the later development of freezing of gait in de novo Parkinson’s disease: An analysis of the PPMI cohort. Parkinsonism Relat. Disord..

[152] Kim R., Jun J.S. (2019). Association of autonomic symptoms with presynaptic striatal dopamine depletion in drug-naive Parkinson’s disease: An analysis of the PPMI data. Auton. Neurosci..

[153] Latourelle J.C., Beste M.T., Hadzi T.C., Miller R.E., Oppenheim J.N., Valko M.P., Wuest D.M., Church B.W., Khalil I.G., Hayete B. (2017). Large-scale identification of clinical and genetic predictors of motor progression in patients with newly diagnosed Parkinson’s disease: A longitudinal cohort study and validation. Lancet Neurol..

[154] Li W., Lao-Kaim N.P., Roussakis A.A., Martín-Bastida A., Valle-Guzman N., Paul G., Loane C., Widner H., Politis M., Foltynie T. (2018). 11 C-PE2I and 18 F-Dopa PET for assessing progression rate in Parkinson’s: A longitudinal study. Mov. Disord..

[155] Lee J.J., Oh J.S., Ham J.H., Lee D.H., Lee I., Sohn Y.H., Kim J.S., Lee P.H. (2016). Association of body mass index and the depletion of nigrostriatal dopamine in Parkinson’s disease. Neurobiol. Aging.

